# Bio-Calcium from Skipjack Tuna Frame Attenuates Bone Loss in Ovariectomy-Induced Osteoporosis Rats

**DOI:** 10.3390/md22100472

**Published:** 2024-10-16

**Authors:** Jirakrit Saetang, Acharaporn Issuriya, Watcharapol Suyapoh, Peerapon Sornying, Krisana Nilsuwan, Soottawat Benjakul

**Affiliations:** 1 International Center of Excellence in Seafood Science and Innovation, Faculty of Agro-Industry, Prince of Songkla University, Hat Yai 90110, Thailand; jirakrit.s@psu.ac.th (J.S.); krisana.n@psu.ac.th (K.N.); 2 Division of Health and Applied Sciences, Faculty of Science, Prince of Songkla University, Hat Yai 90110, Thailand; acharaporn.i@psu.ac.th; 3 Faculty of Veterinary Science, Prince of Songkla University, Hat Yai 90110, Thailand; watcharapol.s@psu.ac.th (W.S.); peerapon.s@psu.ac.th (P.S.)

**Keywords:** postmenopausal, calcium supplement, bone loss, absorption, skipjack tuna

## Abstract

Bio-calcium derived from fish frames may offer several advantages for osteoporosis prevention. This study aimed to evaluate the effects of bio-calcium derived from skipjack tuna frames on bone loss in ovariectomized rats. Tuna bio-calcium was prepared through enzymatic hydrolysis, defatting, bleaching, and grinding processes. The bioavailability of calcium was tested using the Caco-2 cell monolayer model, showing that 13% of tuna bio-calcium was absorbed, compared to 10% for calcium carbonate. Rats were divided into the five following groups: (1) OVX, (2) sham-operated, (3), OVX + estrogen-treated (4) OVX + calcium carbonate-treated, and (5) OVX + tuna bio-calcium-treated. All groups were raised for eight weeks. Tuna bio-calcium was able to increase BV/TV by 26% in the femur and 29% in the tibia, compared to 13% and 17% in the OVX group, respectively. Trabecular thickness in the femur upsurged to 360 µm in the tuna group, while a thickness of 290 µm was observed in the control. Additionally, osteoclast numbers were reduced to 5 N.Oc/mm in the femur and 6 N.Oc/mm in the tibia in the tuna group, compared to 35 and 45 N.Oc/mm in the control. Overall, tuna bio-calcium effectively prevented bone loss and can serve as a promising natural alternative for managing osteoporosis.

## 1. Introduction

Calcium is an important mineral that plays a role in human physiology. Approximately 99% of the body’s calcium is utilized in the skeleton system, providing bone mechanical strength, since it is the major component of hydroxyapatite crystals associated with bone stiffness [1]. This bone mineralization can be maintained through adequate calcium and vitamin D intake, for which 1200 mg/day of calcium is recommended for the daily intake (RDI) of adults. However, when the levels of the circulating calcium are low, parathyroid hormone is released to trigger the demineralization of the bone calcium, leading to the increase in blood calcium supply [2]. Moreover, calcium absorption declines with age, low vitamin D levels, hypochlorhydria, and low estrogen levels [3]. These phenomena happen particularly in the persons who have advanced age and female sex, leading to osteoporosis characterized by low bone mineral density (BMD), bone architecture changes and susceptibility to fractures.

Osteoporosis develops as a skeletal disease with the characteristics of low bone density and strength, causing an increased risk of bone fracture. Postmenopausal women are considered as the high-susceptibility group to this disease, since estrogen deficiency is related to the enhancement of bone demineralization [4,5]. Generally, osteocytes, osteoclasts, and osteoblasts express estrogen receptors on the cell surface, which form homodimers and bind to its ligand and estrogen. This scenario consequently promotes bone remodeling, bone mineralization, and bone formation [6]. On the other hand, the impairment of estrogen production found in postmenopausal women usually results in the increase in bone resorption and osteoporosis.

Due to the importance of calcium in bone health, supplementation with this mineral is recommended in different groups of various ages to improve bone density [7]. Bio-calcium derived from the byproducts of food processing is now recognized as a supplement of choice. This kind of calcium can be alternatively used instead of other types of calcium supplements because of its nutritional values, high bioavailability, and absorption [8,9]. Fishbone bio-calcium powder from the previous work [10,11] had a proportion of 26–30% of calcium with other minerals, including phosphorous, iron, magnesium, etc. This bio-calcium powder showed a small size (17.07–20.29 µm) and was associated with peptides, which is helpful for its solubility and absorption [10]. Some studies also showed that calcium from haddock bones was adequate for calcium supplementation and osteoporosis prevention [12]. Calcium derived from fishbone also showed great benefit over calcium derived from other marine sources, e.g., crustacean shells, which are in the form of calcium carbonate and calcium polyhydroxy phosphate, which are difficult to absorb and increase gastric burden [13].

Skipjack tuna (*Katsuwonus pelamis*) is a species chiefly used for producing canned tuna. During the processing step, half of the skipjack tuna are removed and considered as byproducts, including the tuna frame [14]. Since Thailand is one of the world’s most important canned tuna exporters, the development of bio-calcium from the skipjack tuna frame generated after meat removal has been carried out, facilitated by the abundant raw material and high bioavailability of the product. Although the characteristics of this kind of calcium have been well documented, the role of skipjack tuna bio-calcium on the attenuation of bone loss in osteoporosis has not been elucidated. Therefore, this study aimed to evaluate the effect of skipjack tuna-derived calcium on bone density in ovariectomy-induced osteoporosis rats.

## 2. Results

### 2.1. Bioavailability and Chemical Blood Test

**Figure 1:**
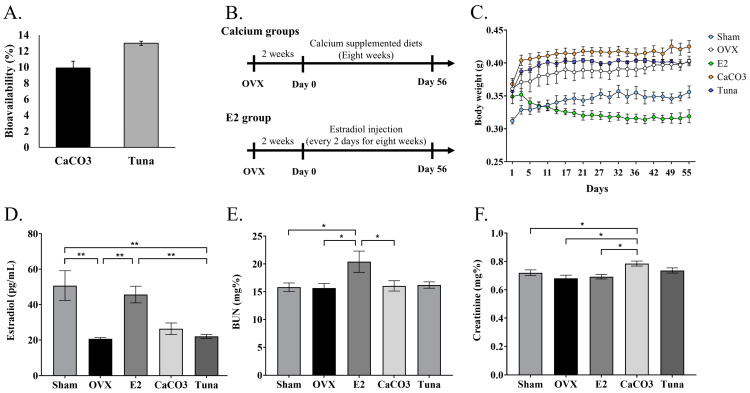
Bioavailability and experimental setup in ovariectomized rats. (**A**) The percentage of calcium absorption measured in Caco-2 monolayer cells comparing tuna bio-calcium and calcium carbonate (CaCO_3_). (**B**) A schematic of the experimental design for calcium supplementation in ovariectomized rats. (**C**) Body weight changes over the 8-week supplementation period across different groups. (**D**) The levels of estradiol in each group measured at the end of the experimental period. (**E**) Blood urea nitrogen (BUN) levels. (**F**) Creatinine levels in all experimental groups. Data are presented as mean ± SEM (*n* = 5). * indicates *p* < 0.05 and ** indicates *p* < 0.01.

The bioavailability of calcium was assessed by the Caco-2 monolayer method. The results demonstrated that 13% of calcium derived from skipjack tuna could be absorbed through the Caco-2 monolayer, while CaCO_3_ showed bioavailability around 10%, which was lower than bio-calcium from tuna (Figure 1A). Therefore, ovariectomy rats were used to elucidate the effect of tuna bio-calcium in attenuating bone loss, as illustrated in Figure 1B. During the calcium supplement period, weight changes were monitored over time, showing two patterns of weight alterations across all groups (Figure 1C). Ovariectomized rats untreated with estradiol showed an increasing trend in weight from around 350–370 g to 390–420 g, while sham and E2 groups displayed consistent weight change and a slight weight loss, respectively, implying the influence of estrogen on the weight change pattern. Moreover, the levels of estradiol in each group of rats underscored the association between estrogen level and weight changes since sham and E2 groups showed the highest levels of estradiol, compared to other groups (*p* < 0.05) (Figure 1D).

Blood urea nitrogen (BUN) and creatinine were also assessed across the different groups in the ovariectomy rat experiment to evaluate kidney function associated with ovariectomy and calcium treatments. The results demonstrated that BUN levels remained relatively stable with the range of 15–20 mg/dL across the groups, although E2 showed slightly higher levels compared to other groups (*p* < 0.05) (Figure 1E). Additionally, creatinine levels also remained constant within normal ranges across all groups, although CaCO_3_ showed slightly increase. This indicated that no significant renal impairment was observed in rats provided with the different treatments.

### 2.2. microCT Analysis of the Bones

**Figure 2:**
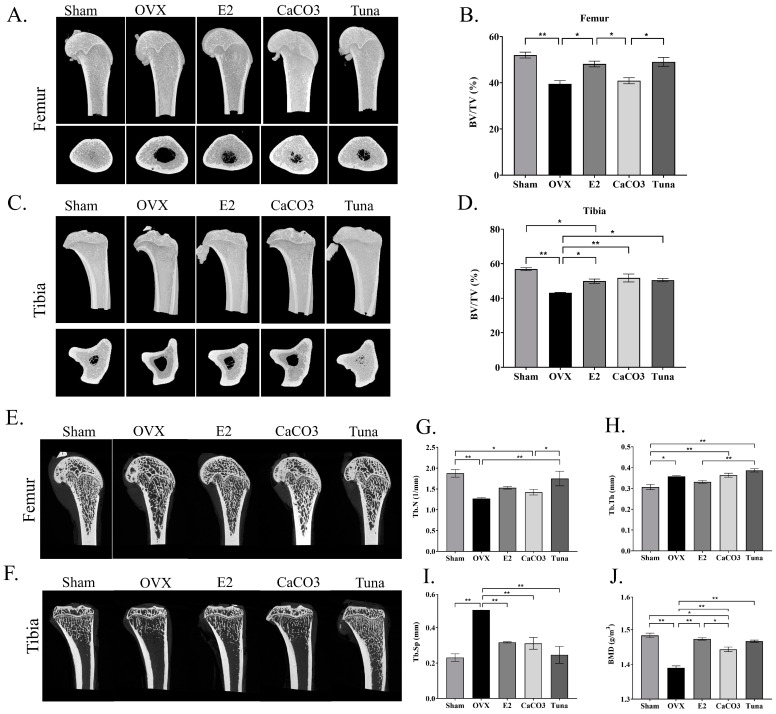
Bone analysis using micro-CT. (**A**) Representative 3D reconstruction images of femur bone across groups. (**B**) The quantification of BV/TV in the femur. (**C**) A 3D reconstruction of the tibial bone. (**D**) The quantification of BV/TV in the tibia. (**E**) Representative images derived from a microCT analysis of femur bone across groups. (**F**) Representative images derived from microCT analysis of tibial bone. (**G**) Trabecular number (Tb.N) of the tibial bone. (**H**) Trabecular thickness (Tb.Th) of the tibial bone. (**I**) Trabecular separation (Tb.Sp) of the tibial bone. (**J**) Bone mineral density (BMD) in femur samples. Data are presented as mean ± SEM (*n* = 4–5). * indicates *p* < 0.05 and ** indicates *p* < 0.01.

Bone microarchitecture was analyzed by microCT imaging to obtain the volume data of bones in the femur and tibia of ovariectomized (OVX) rats. The reconstructed 3D structures of the bones were demonstrated (Figure 2A,C) and the differences in the percentage of bone volume to total volume (BV/TV) between each group were found (Figure 2B,D). Among other groups, OVX rats exhibited the lowest BV/TV values in both femur and tibia (*p* < 0.05) with approximately 39% and 43%, respectively, indicating significant bone loss due to estrogen deficiency from ovariectomy. Interestingly, the sham provided the highest BV/TV values (*p* < 0.05) (around 52% and 55% for femur and tibia, respectively), compared to other treated groups, reflecting normal bone structure. Notably, the CaCO_3_ and tuna groups demonstrated improvements in BV/TV, comparable to the E2 group. These results collectively suggested that both calcium carbonate and tuna bio-calcium supplementation may play significant roles in preserving or enhancing bone density in OVX rats.

The representative 2D images from microCT analysis were also demonstrated (Figure 2E,F). The analysis found the different levels in bone deterioration based on trabecular number (Tb.N), trabecular thickness (Tb.Th), and trabecular separation (Tb.Sp) parameters. The results showed that the OVX group exhibited a significant decrease in Tb.N and Tb.Th, as well as an increase in Tb.Sp (*p* < 0.05), indicating significant bone loss (Figure 2G–I). In contrast, the sham-operated group demonstrated higher Tb.N and Tb.Th, and lower Tb.Sp values (*p* < 0.05). The groups treated with E2, CaCO_3_, and tuna bio-calcium had higher levels in Tb.N and Tb.Th, and lower Tb.Sp (*p* < 0.05), compared to the OVX group. Moreover, the OVX group displayed the lowest BMD values, while the sham group showed higher BMD values (Figure 2J; *p* < 0.05), suggesting healthy bone mass. Notably, the OVX rats subjected to E2 (estrogen), CaCO_3_, and tuna bio-calcium groups exhibited higher BMD, compared to the OVX group (*p* < 005). Interestingly, BMD in the tuna bio-calcium and CaCO_3_ groups were comparable to E2. These findings indicate that both CaCO_3_ and tuna-derived calcium may help mitigate bone loss and restore bone density in ovariectomy rats.

### 2.3. Bone Histomorphometry

**Figure 3:**
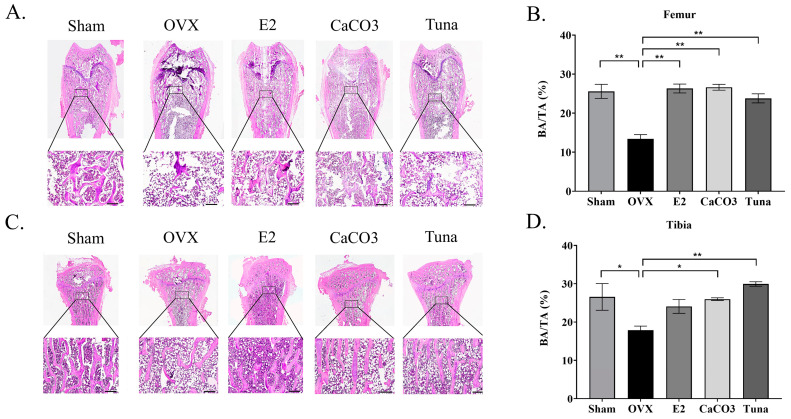
A histomorphometric analysis of bone volume. (**A**) Representative images of bone structure in femur samples. (**B**) A quantitative analysis of bone volume/total volume (BV/TV) in the femur. (**C**) Representative images of tibial bone structure. (**D**) A quantitative analysis of BV/TV in the tibia. Data are shown as mean ± SEM (*n* = 4–5). Original magnification of A and C = ×10 magnification. The scale bar depicts 250 µm. * indicates *p* < 0.05 and ** indicates *p* < 0.01.

To evaluate bone microarchitecture and density, a quantitative histological analysis was conducted. This analysis provided data on BA/TA (bone area/total area), Tb.Th. (trabecular thickness), and Co.Th. (cortical thickness) for the femur and tibia across various treatment groups. The representative images of bone histomorphometry were illustrated in Figure 3A,C. Overall, calcium-treated groups (CaCO_3_ and tuna bio-calcium), the hormone-treated group (E2), and the sham group demonstrated a significantly higher percentage of BA/TA in the femur, ranging from 23 to 26%, compared to the OVX group, which showed around 13% (Figure 3B). No significant difference in the BA/TA value was found in the femur between the sham, E2, CaCO_3_, and tuna groups (*p* > 0.05). The same trend was also observed in the tibia, in which the OVX group demonstrated the lowest BA/TA (around 17%) (*p* < 0.05) compared to other groups (24–29%). However, the BA/TA value of the tibia was slightly higher in tuna group (*p* < 0.05), compared to CaCO_3_, E2, and sham (Figure 3D).

**Figure 4:**
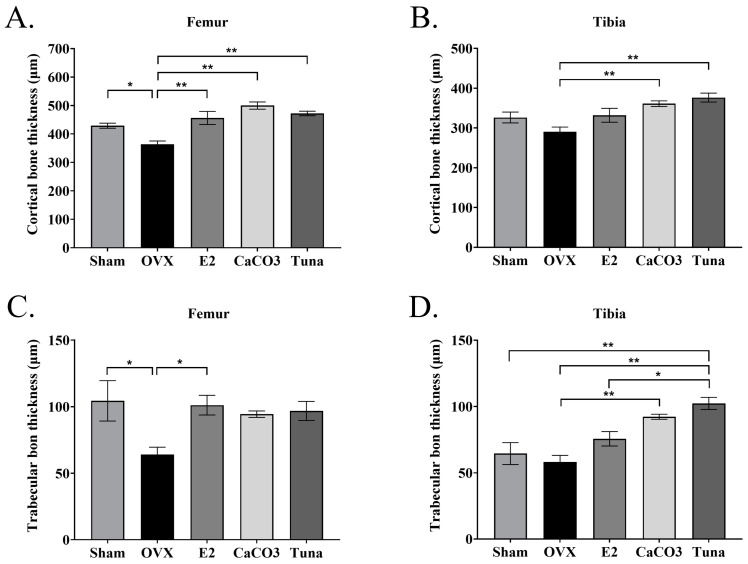
An analysis of trabecular and cortical bone thickness. (**A**) Trabecular thickness (Tb.Th.) in femur samples across all treatment groups. (**B**) Cortical thickness (Co.Th.) in the femur. (**C**) Tb.Th. in the tibia. (**D**) Co.Th. in the tibia. All data are presented as mean ± SEM (*n* = 4–5). * indicates *p* < 0.05 and ** indicates *p* < 0.01.

In addition, trabecular thickness (Tb.Th.) and cortical thickness (Co.Th.) were also evaluated by histomorphometry analysis. For cortical bone thickness, the OVX control group showed the lowest values (*p* < 0.05) (around 363 and 290 µm in femur and tibia, respectively), while the sham, E2, CaCO_3_, and tuna bio-calcium groups exhibited significantly higher cortical bone thickness in both the femur and tibia (*p* < 0.05) (Figure 4A,B). Interestingly, both calcium supplemented groups (CaCO_3_ and tuna bio-calcium) demonstrated the highest values of Co.Th (*p* < 0.05). For trabecular bone thickness, the OVX group also had the lowest trabecular thickness in both the femur and tibia (*p* < 0.05). The other calcium, including sham, E2, CaCO_3_, and tuna bio-calcium showed significantly thicker trabecular bone, with the tuna bio-calcium group having the highest values (*p* < 0.05) (Figure 4C,D).

### 2.4. Osteoclast Analysis

**Figure 5:**
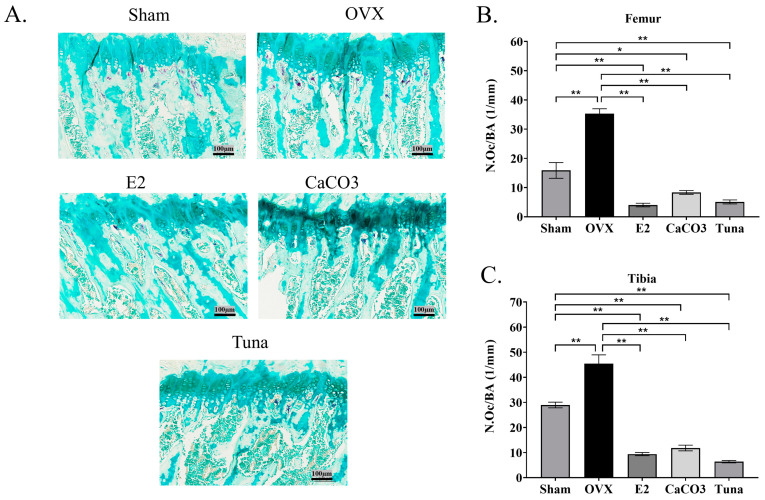
Osteoclast activity analysis using TRAP staining. (**A**) Representative TRAP-stained sections of the femur showing osteoclasts (original magnification = ×20). (**B**) The quantification of osteoclast number per bone area (N.Oc/BA) in the femur. (**C**) The quantification of osteoclast number in the tibia. Data are shown as mean ± SEM (*n* = 4–5). * indicates *p* < 0.05 and ** indicates *p* < 0.01.

The number of osteoclasts was evaluated in the present study since this cell plays a critical role in bone remodeling and homeostasis. Therefore, all groups of rats were examined for the number of osteoclasts that appeared in the femur and tibia. The results demonstrated that osteoclast numbers/bone area (N.Oc/BA) were lower in rats treated with calcium both in the femur and tibia (*p* < 0.05) (Figure 5). As shown in Figure 5A, tartrate-resistant acid phosphatase (TRAP) staining demonstrated the presence of different densities of osteoclast in bone collected from different groups of rats. While the OVX control group showed the highest number of osteoclasts in both the femur and tibia (*p* < 0.05) (around 35 and 45 N.Oc/mm, respectively). The E2, CaCO_3_, and tuna bio-calcium groups all showed significantly lower osteoclast counts, compared to the OVX control and the sham group (*p* < 0.05) (Figure 5B,C). Importantly, tuna bio-calcium group exhibited the lowest osteoclast numbers in both the femur and tibia (*p* < 0.05) (approximately 5 and 6 N.Oc/mm, respectively). These highlighted the potential of bio-calcium, particularly from tuna, in reducing osteoclast cell number and preserving bone structure in estrogen-deficient conditions.

## 3. Discussion

Osteoporosis is a bone disease characterized by decreased bone mass and density, leading to weak and fragile bones that are more susceptible to fractures. Advanced age and female sex are the most important risk factors for osteoporosis, since all bone cells (osteoblasts, osteoclasts and osteocytes) express functional estrogen receptors (ERs), which play a major role in bone metabolism and inhibition [15]. Currently, different regimens have been used to treat osteoporosis, and the supplementation of calcium carbonate and citrate in everyday diets is widely employed. Another prevention and treatment option for osteoporosis is hormone replacement, which induces antiresorptive effects and has been used for decades in menopausal and postmenopausal women; nevertheless, it is accompanied by side effects associated with an increased risk of breast cancer [16].

In the present study, the bio-calcium derived from skipjack tuna frames provided a profound preventive effect on ovariectomy rats. This is in accordance with other studies, demonstrating that fishbone-derived calcium has certain advantages over calcium carbonate supplements or other calcium-rich food. For example, hake fishbone was a good source of calcium, with comparable efficacy to Lithotame (L), a calcium supplement derived from *Lithothamnion calcareum* [17]. A fishbone powder (Phoscalim) and a ray cartilage hydrolysate (Glycollagene) were also reported to be comparable to milk for both short-term calcium absorption and bone resorption [18]. Moreover, tablets made with calcium from haddock bones could prevent osteoporosis in rats [12]. The improvement of bone health found in this study was plausibly due to the high bioavailability of bio-calcium. The association between calcium and peptides, as well as the small crystal size, provided the augmented bioavailability [19,20]. Several studies have shown that peptide-bound calcium has higher bioavailability than commonly used calcium supplements, such as calcium carbonate [21]. Additionally, the presence of peptides and glycosaminoglycans in fishbones may have a positive effect on osteoblastic differentiation, which further promoted the bone-protective effects of the tuna-derived bio-calcium [22,23].

The increased bone volume/total volume (BV/TV) and trabecular thickness were observed in the tuna bio-calcium group, compared to the control group. It was postulated that the activation of the calcium-sensing receptor by skipjack tuna bio-calcium occurred. Calcium serves as a crucial activator of osteoblasts via the calcium-sensing receptor (CaSR) [24]. The activation of CaSR by extracellular calcium stimulates osteoblast proliferation, differentiation, and mineralization through the phosphatidylinositol 3-kinase (PI3K)/Akt and mitogen-activated protein kinase (MAPK) pathways [24,25]. Furthermore, bio-calcium from tuna is rich in phosphorus and magnesium, which might synergistically enhance osteoblast activity, and promote the deposition of hydroxyapatite in bone [24].

In addition to the improvement of bone microstructure, the effects of bio-calcium supplementation observed in this study were consistent with the known effects of calcium on the bone remodeling process in terms of influencing osteoclasts’ number and activity [23,26]. Normally, calcium plays a remarkable role in maintaining the balance between bone resorption by osteoclasts and bone formation by osteoblasts, a process regulated by estrogen, parathyroid hormone (PTH), and vitamin D [27]. The estrogen deficiency induced by ovariectomy in the rat model led to increased osteoclast activity and bone resorption [27]. However, the present study observed the lower numbers of this cell in the ovariectomized rats treated with tuna bio-calcium group. This might be due to the higher bioavailability of bio-calcium from skipjack tuna, since it was reported that the supplementation of calcium could downregulate the RANKL/RANK pathway, which reduced the differentiation and activity of osteoclast [26]. The reduced number of osteoclasts in the femur and tibia in this study may be attributed to the inhibition of RANKL in response to increased extracellular calcium from the bio-calcium supplement. This would help maintain bone mass by reducing bone resorption, a crucial process in the progression of osteoporosis [9].

Although the effect of tuna bio-calcium in osteoporosis found in this study was comparable to CaCO_3_, bio-calcium from the fish frame still exhibited lower adverse effects due to its high bioavailability. Notably, calcium carbonate is well known for its poorly absorbed and causing gastrointestinal side effects, including bloating, gas, and constipation [13]. These issues arise because calcium carbonate requires stomach acid for absorption, which can lead to indigestion and discomfort, especially in individuals with low stomach acid, such as the elderly [28]. In contrast, bio-calcium from tuna frames exhibits higher bioavailability due to its peptide-bound structure and smaller particle size, which enhances solubility and absorption as shown in Caco-2 monolayer model. Studies indicated that peptide-bound calcium is more efficiently absorbed in the intestines compared to inorganic calcium forms like CaCO_3_ [20,21]. The presence of bioactive peptides and glycosaminoglycans in bio-calcium may also support gastrointestinal health by promoting gut barrier function and reducing inflammation [20]. These findings suggested that bio-calcium from the tuna frame could offer a safer alternative to traditional calcium carbonate supplements, particularly for individuals with gastrointestinal sensitivities or impaired calcium absorption.

## 4. Materials and Methods

### 4.1. Skipjack Tuna Bio-Calcium Preparation

Skipjack tuna frames were cut into small pieces of 4–5 cm by using a sawing machine. Before hydrolysis, the prepared frames were mixed with water at a ratio of 1:2 (*w*/*v*) and the pH was adjusted to 8.0 using 10 M sodium hydroxide solution. The temperature was set to 60 °C, before adding alcalase at the final concentration of 3% (by frame weight). The reaction was stirred continuously for 3 h. Then, the protein-free bones were separated by filtering with a sieve and washed with clean water. The prepared bones were then dried at 60 °C for 6 h in a hot-air oven (Memmert UF30, Schwabach, Germany). Thereafter, fat was then removed by soaking the dried tuna bones in hexane at a ratio of 1:10 (*w*/*v*) for 1 h at 25–30 °C, before drying to eliminate the solvent.

For decolorization, the defatted bones were soaked continuously in 2.5% hydrogen peroxide solution (*v*/*v*) at a ratio of 1:10 (*w*/*v*) for 1 h at 25–30 °C. The solution was changed every 30 min, followed by the washing step using water at a 1:10 (*w*/*v*) to soak the bones for 30 min. The decolorized bones were then dried in a hot-air oven at 60 °C for 6 h. Tuna bone powder was prepared by grinding the dried fishbones using a high-speed grinder, followed by fine grinding with a ball mill (PM 100, Retsch GmbH, Haan, Germany) using steel balls with a diameter of 20 mm. The ratio of sample to steel balls was 1:2 (*v*/*v*). The grinding step was carried out for 2.5 h. Finally, the powders were sieved using a 75 µm screen with the aid of a sieving machine (Endecotts, London, UK) to obtain a tuna bio-calcium powder.

### 4.2. Preparation of Digested Bio-Calcium

To simulate the digestion of bio-calcium in humans, an in vitro digestion system was used. Briefly, 75 mg of bio-calcium was mixed with 50 mL of 5 mM HCl-KCl buffer at pH 1.5, before the addition of 2.5 mL of 40 mg/mL pepsin solution. The mixture was incubated at 37 °C with constant agitation at 80 rpm for 60 min. Subsequently, the pH was adjusted to 6.8 using NaHCO_3_, and a mixture containing 500 µL of bile, 500 µL of pancreatic enzyme (pancreatin (80 mg/mL), and bile extract (40 mg/mL) in 10 mM Tris-HCl buffer at pH 8.2) was added. The system was incubated at 37 °C for 3 h and shaken at 80 rpm. The reaction was stopped by heating the solution at 95 °C for 10 min, and then centrifuged at 7000× *g* for 10 min. The supernatant was collected for an analysis of the calcium content by using Flame Atomic Absorption Spectrometry (FAAS) and was also used for further analysis.

### 4.3. Bioavailability Evaluation of Skipjack Tuna Derived Bio-Calcium

The bioavailability of bio-calcium from skipjack tuna bone was tested by evaluating the ability of digest to be transported through Caco-2 monolayer cells. Caco-2 cells were maintained in Dulbecco’s modified Eagle’s medium (DMEM) with 10% fetal bovine serum at 37 °C in a CO_2_ incubator. For the experiment, the cells were prepared at 5.0 × 10^4^ cells/well with 1.5 mL of medium on the upper chamber of 6-transwell plates with 0.4-micrometer pores. The lower chambers were filled with 2 mL of serum-free medium. Cells were cultured for 25 days to allow the differentiation into intestinal-like cells. During this time, the medium was changed every 2 days, and the integrity of the cells was assessed every 3 days by measuring the Transepithelial Electric Resistance (TEER) using a TEER Voltohmmeter (Millicell-ERS, EMD Millipore Corporation, Burlington, MA, USA).

After 25 days, the medium was changed to serum-free medium (1.5 mL), and 50 µL of digested bio-calcium was added. The system was incubated at 37 °C for 4 h, and the TEER value was measured again to confirm the integrity of the monolayer cell. The medium from the lower chamber was then collected and measured for the concentration of calcium using FAAS (Flame Atomic Absorption Spectrometry). The bioavailability of the bio-calcium was calculated using the following formulation:

Bioavailability (%) = (Calcium amount in the lower chamber/Total initial calcium amount) × 100(1)

### 4.4. Animal Model

Female Sprague Dawley rats (*n* = 30, age: 6 month old, mean body weight: 350 ± 10 g) were obtained from Nomura Siam International (Bangkok, Thailand), and maintained in the Southern Laboratory Animal Facility, Faculty of Science, Prince of Songkla University. The housing conditions included a temperature of 22 ± 3 °C, a relative humidity of 30 ± 10%, and a 12 h light–dark cycle. The animals were sham-operated or bilaterally ovariectomized at day 0 after 7 days of quarantine. Bilateral OVX was performed using the dorsal method. Rats were anesthetized by an intramuscular injection of xylazine (4 mg/kg) and Zoletil (30 mg/kg). Moreover, ampicillin (100 mg/kg; intramuscular injection) and carprofen (0.5 mg/kg; subcutaneous injection) were used to treat rats daily for 4 days after ovariectomy.

The ovariectomized rats were randomly divided into five groups (five individuals each), including no removal of ovaries (sham), OVX+ no treatment (OVX), OVX + estradiol (E2), OVX + CaCO_3_ (CaCO_3_), and OVX + bio-calcium (tuna). All types of calcium supplementation were started on day 14 after ovariectomy and all the experiments were continued for eight weeks. Group I (sham) comprised rats from whom only a piece of fat around the ovaries was excised, and they were provided with the standard diet. Group II (OVX) was provided with the standard diet. Group III (E2) was injected with estradiol (10 μg/kg) every two days subcutaneously and received the standard diet. Group IV (CaCO_3_) received a pellet diet containing CaCO_3_. Group V (tuna) received the diet supplemented with skipjack tuna bone-derived bio-calcium. For the calcium supplement groups (Group IV and Group V), a total calcium of 1.5% was included in the diet. Over 8 weeks, the body weight of the animals was recorded every 3–4 days. The animal experiments were approved under the guidelines and permission of Animal Care Committee of Prince of Songkla University, approval number AR3030/2022.

### 4.5. Biochemical Analysis

Rats from all groups were then sacrificed after 8 weeks of treatment. Blood was collected by cardiac puncture under total anesthesia by overdose pentobarbital sodium (150 mg/kg, i.p.) and then centrifuged to obtain heparin plasma. Blood urea nitrogen, estrogen, and creatinine were measured by spectrophotometric methods. The plasma samples were stored at −80 °C until assayed.

### 4.6. Quantitative 3D Analyses of Bone

After 8 weeks of treatment, the left tibia and femur of rats were dissected. Both femoral bones and the tibia were stored at −20 °C until use. The analysis of the 3D structure of bone tissues was conducted in a dry state using microcomputed tomography (micro-CT, SkyScan 1173, Bruker, MA, USA) with a 14 μm voxel resolution, 80 kV voltage, and 100 μA current of image acquisition. Bone structures were analyzed for the following parameters: relative bone volume (%), trabecular thickness, trabecular separation, trabecular number, and bone mineral density. The analysis of cortical bone was started at 11.8 mm from the end of the growth plate (distal end) and moving to 2 mm at femoral midshaft. For the trabecular bone, the analysis was carried out at the region between 2.9 mm from the end of the growth plate (distal end) to 4.9 mm.

### 4.7. Histomorphological Study

The right tibia and femur of rats were collected and fixed in 10% buffered formalin for 72 h before decalcification. Briefly, bone samples were decalcified with 10% ethylenediaminetetraacetic acid (EDTA) at room temperature (RT) for 2–3 weeks [29]. Decalcified samples were rinsed in tap water and further processed by routine histological techniques. Both the femur and tibia were stained by hematoxylin and eosin (H&E) before visualizing with the aid of a VDO capture digital camera (ECLIPSE Ni-U) (Nikon, Tokyo, Japan. Bone parameters, including bone volume/total volume (BV/TV), trabecular thickness (Tb. Th), and cortical bone thickness (Co.Th), were then determined in all samples. All parameters were measured using the Fiji ImageJ software (version 2.99.0/1.53t; NIH, Bethesda, MD, USA). BoneJ library was used for analyzing standard bone BV/TV parameter from 2D images [30].

### 4.8. Osteoclast Analysis

Formalin-fixed paraffin embedded sections were melted in a microwave oven at 60 °C for 15 min. Samples were then deparaffinized in xylene and re-hydrated in ethanol. The prepared tissues were then stained for tartrate-resistant acid phosphatase (TRAP) using TRACP and ALP Assay Kit (Takara bio, Shiga, Japan) to identify osteoclast cells. The stained cells were visualized and counted by Image J software (National Institutes of Health, Bethesda, MD, USA).

### 4.9. Statistical Analysis

The in vitro experimental data were presented as mean ± standard deviation (SD). The data from the animal study were expressed as mean ± SEM. The statistical significance was analyzed using a parametric one-way ANOVA followed by Tukey’s honestly significant difference test. A *p* value < 0.05 was considered statistically significant. SPSS software 20.0 (SPSS Inc., Chicago, IL, USA) was used for all analyses.

## Data Availability

The data presented in this study are available on request from the corresponding author.
